# Prevalence of anxiety, depression, and post-traumatic stress disorder among Omani children and adolescents diagnosed with cancer: a prospective cross-sectional study

**DOI:** 10.1186/s12885-024-12272-z

**Published:** 2024-04-23

**Authors:** Laila S. Al-Saadi, Moon Fai Chan, Amal Al Sabahi, Jalila Alkendi, Nawal Al-Mashaikhi, Hana Al Sumri, Amal Al-Fahdi, Mohammed Al-Azri

**Affiliations:** 1 Department of Family Medicine and Public Health, College of Medicine and Health Sciences, Sultan Qaboos University, Postal Code 123, Al Khoud, Al Khoud, Muscat, PO Box 38, Oman; 2 National Oncology Centre, Royal Hospital, Bawshar, Muscat Oman; 3 Department of Child Health, Sultan Qaboos University Hospital, Al Khoud, Muscat, Oman; 4 Sultan Qaboos Comprehensive Cancer Care and Research Centre (SQCCCRC), Al Khoud, Muscat, Oman

**Keywords:** Anxiety, Depression, Post-traumatic stress disorder, Children, Adolescents, Cancer, Oman

## Abstract

**Background:**

Children and adolescents diagnosed with cancer often experience psychological distress, encompassing anxiety, depression, and post-traumatic stress disorder (PTSD). This study aimed to evaluate the prevalence of these conditions among Omani children and adolescents diagnosed with cancer, alongside identifying contributing factors.

**Methods:**

A prospective cross-sectional study was conducted from October 2021 to June 2023 among a cohort of Omani children and adolescents (6–18 years old) diagnosed with cancer at three primary cancer referral centres in Oman. Validated Arabic-language versions of the Screen for Child Anxiety Related Disorders, the Center for Epidemiologic Studies Depression Scale for Children, and the Impact of Event Scale-Revised instruments were used to assess symptoms of anxiety, depression, and PTSD, respectively. An initial assessment (T1) was undertaken within the first 3 months of diagnosis, followed by a second assessment (T2) 3–6 months later.

**Results:**

Of 113 eligible participants, 101 agreed to participate in the study (response rate: 95.6%), with 92 (91.0%) completing both assessments and included in the final analysis. Prevalence rates of anxiety, depression, and PTSD decreased from 43.5%, 56.5%, and 32.6%, respectively, at T1, to 38.0%, 35.9%, and 23.9% at T2. All average scores were below diagnostic cut-off points, except for the depression score at T1. Anxiety and depression scores decreased significantly (*p* = 0.043 and 0.001, respectively) between T1 and T2, as did the overall prevalence of depression (*p* = 0.004). At T1, linear regression analysis showed significant correlations between anxiety scores and the child’s age and PTSD score (*p* < 0.05); these variables were also correlated with depression scores (*p* ≤ 0.001). At T2, significant correlations were observed between anxiety scores and the child’s age and PTSD scores (*p* < 0.001). At both T1 and T2, anxiety, depression, and PTSD scores remained significantly correlated (*p* < 0.001).

**Conclusions:**

Omani children and adolescents recently diagnosed with cancer exhibit a high prevalence of anxiety, depression, and PTSD over time. Age-appropriate communication, ongoing support, and mental health services are recommended to help this patient group cope with their diagnosis and manage their emotional wellbeing. There is a need for future research to determine the effectiveness of specific psychological interventions in reducing the frequency of these disorders.

## Background

Cancer in childhood and adolescence ranked as the sixth leading contributor to the total global cancer burden in 2019 [1]. An estimated 429,000 individuals under 19 years of age are diagnosed with cancer every year, with 141–185 cases per million reported worldwide [2, 3]. Approximately 100,000 children and adolescents die annually from cancer, with the vast majority of deaths (90%) occurring in low- and middle-income countries (LMICs) [1]. Furthermore, those diagnosed with cancer in LMICs have a low five-year survival rate of 30%, in stark contrast to high-income countries where survival rates exceed 80% due to significant advances in cancer treatment [2, 3].

In Arab countries, over 18,000 children below the age of 15 years are diagnosed with cancer every year, with annual incidence rates ranging from 7.5 to 12.8 cases per 100,000 children, although variations may be due to differences in registration accuracy [4]. In Oman, approximately 31% of the total population is under 19 years of age [5]. In 2019, a total of 2,307 patients were diagnosed with cancer, of which 2,089 patients (91.5%) were of Omani nationality and 124 (5.9%) comprised children aged 0 to 14 years [6]. However, the anticipated total number of annual cancer diagnoses is projected to rise to 8,549 by the year 2040 [7].

Anxiety, depression, and post-traumatic stress disorder (PTSD) are frequent in children and adolescents with cancer, with pooled prevalence rates of 13.92%, 20.43%, and 20.90%, respectively [8]. Recent research underscores a higher incidence of anxiety and depression in paediatric cancer patients and the heightened vulnerability of this demographic to post-traumatic stress symptoms, emphasising the need for a nuanced understanding of emotional challenges throughout the cancer diagnosis, treatment, recovery, and survivorship journey [9–12]. In itself, a diagnosis of cancer, along with accompanying physical symptoms and treatment side-effects, can lead to excessive tension, discomfort, and fear of death [8, 13]. Symptoms of depression, including low mood, despair, guilt, and loss of interest in usual activities, may also challenge patients’ ability to function and adhere to treatment [14].

As Oman continues to make significant improvements in healthcare delivery and medical treatment, cancer survival rates among children and adolescents have improved. However, the emotional toll of a cancer diagnosis cannot be underestimated, and an understanding of these psychological repercussions is crucial as an essential indicator of patients’ well-being to ensure the provision of comprehensive oncologic care [8]. Indeed, it has been found that that the activity of making jewelry from beads was effective in reducing the state and trait anxiety levels of children with cancer [15]. Our study therefore aimed to identify the prevalence of anxiety, depression, and PTSD among Omani children and adolescents diagnosed with cancer and their associated factors, and to describe changes occurring over time.

## Methods

### Study design and setting

A cross-sectional study was conducted targeting all Omani children and adolescents aged six to 19 years diagnosed with any type of cancer between 1st October 2021 and 30th June 2023. The study was conducted at the National Oncology Centre (NOC) of the Royal Hospital, the Paediatric Haematology Unit of the Sultan Qaboos University Hospital (SQUH), and the Sultan Qaboos Comprehensive Cancer Care and Research Centre (SQCCCRC). These centres, located in Muscat, the capital city of Oman, serve as the primary referral cancer centres providing integrated care for cancer patients throughout Oman.

### Recruitment of participants

Participants were recruited during their visits to either the outpatient clinics of the three referral centres or upon admission to the oncology/haematology wards. Children and adolescents who were non-Omani or had cognitive and behavioural disorders (as documented in their medical records) were excluded from the study.

### Data collection

An Arabic version of the Screen for Child Anxiety Related Disorders (SCARED) tool was used to screen for anxiety symptoms over the past three months [16, 17]. It consists of various questions or items related to anxiety symptoms, and individuals are typically asked to respond based on their experiences which is valuable for understanding the child’s mental health status over a recent period [16]. This child self-report instrument includes 41 items scored on a 3-point scale (from 0 to 2), yielding five factors matching classifications outlined in the Diagnostic and Statistical Manual of Mental Disorder, fifth edition (DSM-IV) [16]. Overall, a total SCARED score of ≥ 25 may indicate the presence of an anxiety disorder, while scores of > 30 are more specific to anxiety. According to a validation study, internal consistency (Cronbach’s α) for the translated tool is 0.91, ranging between 0.65 and 0.89 for individual subscales [16].

Depressive symptoms were assessed using an Arabic version of the Center for Epidemiologic Studies Depression Scale for Children (CES-DC) [18, 19]. It consists of a series of questions that ask about various feelings and behaviours associated with depression, such as sadness, irritability, changes in appetite or sleep patterns, and feelings of worthlessness [19]. Respondents rate how often they have experienced each symptom over a specific period, typically within the past week [19]. This self-report scale consists of 20 items scored on a 4-point scale (from 0 to 3), for a total score ranging from 0 to 60, with higher scores more indicative of depression [18]. The cut-off CES-DC score is 15, with scores of 15–60 considered indicative of significant symptoms of depression. The Arabic version of the CES-DC has previously demonstrated high internal consistency (Cronbach’s α = 0.90) [18].

An Arabic version of the Impact of Event Scale-Revised (IES-R) was used to measure symptoms of post-traumatic stress [20, 21]. The IES-R is a self-report scale designed to assess current subjective distress for any major life event in children, adolescents, and adults, assessing the core symptom cluster of avoidance, intrusion, and hyperarousal [20, 21]. The tool has been also used to evaluate the extent of distress experienced by individuals who have been exposed to a traumatic event such as accidents, natural disasters, combat, or other life-threatening situations [21]. The scale helps clinicians and researchers understand the psychological impact of these events on individuals [20]. The tool consists of 22 items scored on a 5-point scale (from 0 to 4), of which 14 items correspond directly to symptom criteria outlined in the DSM-IV. Total scores range from 0 to 88, with a cut-off IES-R score of 33 and above indicative of a probable diagnosis of PTSD [20, 21]. According to previous research, the Arabic version of the self-report IES-R scale has demonstrated acceptable internal consistency (Cronbach’s α = 0.94) [20].

Arabic versions of the SCARED, CES-DC, and IES-R instruments were administered twice to assess for symptoms of anxiety, depression, and PTSD, respectively. The first assessment (T1) was conducted at any time within the first 3 months of diagnosis, while the second assessment (T2) was conducted 3 to 6 months after T1. Research assistants administered the questionnaire to participants aged 6 to 12 years, while the instruments were self-administered by participants aged 12 years or older. In both cases, a research assistant remained available to clarify any questions that the participants might have had during completion. Additional sociodemographic and clinical information was recorded by the researchers based on data gathered from the participants’ medical records or elicited from the children’s parents or primary caregivers at T1. Cancer risk was estimated based on the participant’s age at diagnosis, disease stage, tumour histology, MYCN status (amplified versus nonamplified), and tumour cell ploidy status [22].

### Statistical analysis

Statistical analysis was performed using SPSS Statistics Software for Windows, version 23 (IBM Corp., Armonk, NY). Descriptive statistics, including percentages, frequencies, means, and standard deviations, were used to delineate the participants’ sociodemographic and clinical characteristics, as well as their average anxiety, depression, and PTSD scores. Paired t-tests and McNemar’s tests were utilised to assess differences in average anxiety, depression, and PTSD scores between the two time points. Analysis of variance and independent sample t-tests were employed to assess variations between the dependent variable (average anxiety, depression, or PTSD scores) and independent variables (sociodemographic and clinical characteristics). Pearson’s Chi-squared test was applied to explore associations between psychological outcomes and sociodemographic and clinical characteristics. Linear regression models were used to investigate correlations between sociodemographic and clinical characteristics and average anxiety, depression, and PTSD scores. The researchers adhered to a significance level of 5% throughout the analysis.

## Results

### Characteristics of the participants

Out of the 113 Omani children and adolescents diagnosed with cancer during the study period, 101 agreed to participate, yielding a response rate of 95.6%. However, only 92 participants (91.0%) completed both T1 and T2 assessments and were included in the final analysis. Of these, 83 (90.2%) received a new diagnosis of cancer, while nine (9.8%) had suffered relapses. The mean age was 11.4 ± 3.6 years, with a median of 11.0 years. Most participants (*n* = 62; 67.4%) were children (aged 6–12 years). Males (*n* = 52; 56.5%) outnumbered females (*n* = 40; 43.5%). Leukaemia was the most frequent diagnosis (*n* = 38; 41.3%), with most participants receiving chemotherapy as the sole form of treatment (*n* = 56; 60.9%). Most participants were assessed within the first month of diagnosis (*n* = 66; 71.7%), with their cancer diagnosis not being disclosed to them (*n* = 65; 70.7%) [see Table 1].

**Table 1:** Baseline sociodemographic and clinical characteristics of Omani children and adolescents diagnosed with cancer (*N* = 92)

Characteristic	N (%)
**Gender**	
Female	40 (43.5)
Male	52 (56.5)
**Age, years**	
Mean ± SD	11.4 **±** 3.6
Median (range)	11.0 (6.0–18.0)
**Age group**	
Children	62 (67.4)
Adolescents	30 (32.6)
**Hospital**	
NOC	60 (65.2)
SQUH	22 (23.9)
SQCCCRC	10 (10.9)
**Diagnosis**	
Leukaemia	38 (41.3)
Lymphoma	25 (27.2)
Solid tumour	23 (25.0)
Brain tumour	6 (6.5)
**Time since diagnosis, months**	
< 1	66 (71.7)
1–2	19 (20.7)
2–3	7 (7.6)
**Type of cancer diagnosis**	
Relapse	9 (9.8)
New	83 (90.2)
Cancer risk*	
Not applicable	6 (6.5)
Standard/low	18 (19.6)
Intermediate	26 (28.3)
High	42 (45.7)
**Cancer stage**	
Not applicable	39 (42.4)
I	7 (7.6)
II	8 (8.7)
III	18 (19.6)
IV	20 (21.7)
**Treatment type**	
Chemotherapy alone	56 (60.9)
Chemotherapy and radiotherapy	12 (13.0)
Chemotherapy and surgery	9 (9.8)
Chemotherapy, radiotherapy, and surgery	10 (10.9)
Chemotherapy and transplant	5 (5.4)
**Aware of diagnosis**	
Yes	27 (29.3)
No	65 (70.7)

Abbreviations: SD: Standard Deviation, NOC: National Oncology Centre, SQUH: Sultan Qaboos University Hospital, SQCCCRC: Sultan Qaboos Comprehensive Cancer Care and Research Centre. *Estimated based on the participant’s age at diagnosis, disease stage, tumour histology, MYCN status (amplified versus nonamplified), and tumour cell ploidy status [21]

### Prevalence of anxiety, depression, and PTSD

Overall, 40 (43.5%) and 35 (38.0%) participants exhibited anxiety symptoms at T1 and T2, respectively, while 52 (56.5%) and 33 (35.9%) reported depressive symptoms and 30 (32.6%) and 22 (23.9%) had probable diagnoses of PTSD. There was a significant reduction in the prevalence of depression between T1 and T2 (*p* = 0.004). Similarly, average scores decreased significantly for both anxiety (23.7 ± 10.6 vs. 21.7 ± 11.0; *p* = 0.043) and depression (17.67 ± 10.7 vs. 13.6 ± 8.9; *p* = 0.001) during this interval [see Table 2].

**Table 2:** Prevalence of anxiety, depression, and post-traumatic stress disorder* over time among Omani children and adolescents diagnosed with cancer (*N* = 92)

	First assessment (T1)	Second assessment (T2)	Statistics†	*p* value
**Prevalence, n (%)**				
**Anxiety**				
Yes	40 (43.5)	35 (38.0)	8.633	0.473
No	52 (56.5)	57 (62.0)		
**Depression**				
Yes	52 (56.5)	33 (35.9)	2.155	0.004‡
No	40 (43.5)	59 (64.1)		
**PTSD**				
Yes	30 (32.6)	22 (23.9)	26.248	0.096
No	62 (67.4)	70 (76.1)		
**Average score, mean ± SD**				
Anxiety	23.7 ± 10.6	21.7 ± 11.0	2.050	0.043‡
Depression	17.7 ± 10.7	13.6 ± 8.9	3.472	0.001‡
PTSD	23.9 ± 18.5	21.3 ± 16.8	1.619	0.109

Abbreviations: PTSD: Post-Traumatic Stress Disorder, SD: Standard Deviation. *The presence of anxiety, depression, and PTSD symptoms was assessed using validated Arabic versions of the Screen for Child Anxiety Related Disorders, Center for Epidemiologic Studies Depression Scale for Children, and Impact of Event Scale-Revised instruments, respectively. Scores of ≥ 25, ≥15, and ≥ 33, respectively, were considered indicative of an anxiety disorder, depressive symptoms, and a probable diagnosis of PTSD [15–20]. †Calculated using either McNemar’s test or a paired t-test, as appropriate. ‡Considered statistically significant at *p* ≤ 0.05

### Factors associated with anxiety, depression, and PTSD

At T1, the univariate analysis revealed a statistically significant increase in average scores for anxiety (*p* = 0.005), depression (*p* < 0.001), and PTSD (*p* < 0.001) as the child’s age advanced. Adolescents (aged 13–18 years) demonstrated significantly increased anxiety (*p* = 0.041), depression (*p* = 0.012), and PTSD (*p* = 0.001) scores compared to children (aged 6–12 years). Participants identified as having a high risk of cancer exhibited significantly increased PTSD scores (*p* = 0.001), while those aware of their cancer diagnosis showed significant increases in both anxiety (*p* = 0.003) and PTSD (*p* = 0.004) scores. Increased anxiety scores correlated with significant increases in both depression (*p* < 0.001) and PTSD (*p* < 0.001) scores; similarly, increased depression scores were associated with higher anxiety (*p* < 0.001) and PTSD (*p* < 0.001) scores, while elevated PTSD scores were associated with significant increases in both anxiety (*p* < 0.001) and depression (*p* < 0.001) scores [see Table 3].

**Table 3:** Associations between selected sociodemographic, clinical, and psychological factors and mean anxiety, depression, and post-traumatic stress disorder scores* at first investigation (T1) among Omani children and adolescents diagnosed with cancer (*N* = 92)

Characteristic	Anxiety score			Depression score			PTSD score		
	Mean ± SD	Statistics†	*p* value	Mean ± SD	Statistics†	*p* value	Mean ± SD	Statistics†	*p* value
**Gender**									
Female	24.9 ± 11.4	0.889	0.376	15.7 ± 9.8	-1.598	0.114	23.4 ± 17.4	-0.242	0.810
Male	22.9 ± 10.0			19.2 ± 11.3			24.4 ± 19.5		
**Age‡**	-	0.288	0.005^	-	0.383	< 0.001^	-	0.436	< 0.001
**Age group**									
Children	22.2 ± 10.5	-2.094	0.041^	15.7 ± 10.6	-2.576	0.012^	19.5 ± 17.8	-3.470	0.001^
Adolescents	27.0 ± 10.2			21.7 ± 10.2			33.0 ± 16.8		
**Diagnosis**									
Leukaemia	22.5 ± 9.6	0.330	0.804	17.9 ± 10.2	0.055	0.983	23.9 ± 16.8	2.054	0.112
Lymphoma	24.1 ± 11.4			16.9 ± 11.4			17.6 ± 16.5		
Solid tumour	25.0 ± 11.4			18.0 ± 11.5			28.3 ± 21.3		
Brain tumour	25.3 ± 12.0			18.0 ± 10.4			33.8 ± 21.4		
**Time since diagnosis, months**									
< 1	23.1 ± 10.0	1.250	0.291	17.6 ± 11.1	0.473	0.625	21.6 ± 18.4	2.505	0.087
1–2	27.0 ± 13.5			16.7 ± 9.9			27.5 ± 18.2		
2–3	21.1 ± 5.9			21.3 ± 9.8			36.3 ± 15.9		
**Type of cancer diagnosis**									
Relapse	25.1 ± 12.3	0.410	0.683	20.1 ± 12.0	0.714	0.477	32.1 ± 13.7	1.402	0.164
New	23.6 ± 10.5			17.4 ± 10.6			23.1 ± 18.8		
**Cancer risk**¶									
Not applicable	19.7 ± 8.2	0.972	0.410	15.0 ± 6.8	2.234	0.090	9.5 ± 3.8	6.143	0.001^
Standard	21.1 ± 10.2			15.6 ± 11.2			16.0 ± 17.4		
Intermediate	23.9 ± 11.2			14.8 ± 10.0			20.0 ± 18.8		
High	25.3 ± 10.7			20.8 ± 10.9			31.8 ± 16.9		
**Cancer stage**									
Not applicable	22.1 ± 9.7	0.601	0.663	17.5 ± 10.4	2.326	0.063	23.4 ± 16.8	2.122	0.085
I	26.3 ± 10.9			15.0 ± 11.4			19.0 ± 18.2		
II	27.5 ± 13.2			15.3 ± 12.0			16.6 ± 16.2		
III	24.7 ± 11.9			13.8 ± 8.6			19.6 ± 18.1		
IV	23.5 ± 10.4			23.4 ± 11.2			33.5 ± 20.9		
**Treatment type**									
Chemotherapy alone	21.6 ± 9.2	1.527	0.201	16.5 ± 11.3	0.939	0.445	20.2 ± 17.9	1.876	0.122
Chemotherapy and radiotherapy	27.3 ± 11.1			17.3 ± 9.3			25.7 ± 19.5		
Chemotherapy and surgery	26.9 ± 12.2			22.1 ± 9.2			28.0 ± 19.3		
Chemotherapy, radiotherapy, and surgery	25.7 ± 13.6			17.9 ± 11.3			34.0 ± 19.7		
Chemotherapy and transplant	29.0 ± 12.6			23.6 ± 9.0			34.0 ± 9.4		
**Aware of diagnosis**									
Yes	28.7 ± 11.2	3.053	0.003^	19.1 ± 10.1	0.825	0.411	32.5 ± 16.1	2.973	0.004^
No	21.7 ± 9.7			17.1 ± 11.0			20.4 ± 18.4		
**Psychological distress**									
Anxiety‡		-	-		0.369	< 0.001^		0.475	< 0.001^
Depression‡		0.369	< 0.001^		-	-		0.667	< 0.001^
PTSD‡		0.475	< 0.001^		0.667	< 0.001^		-	-

Abbreviations: PTSD: Post-Traumatic Stress Disorder, SD: Standard Deviation. *The presence of anxiety, depression, and PTSD symptoms was assessed using validated Arabic versions of the Screen for Child Anxiety Related Disorders, Center for Epidemiologic Studies Depression Scale for Children, and Impact of Event Scale-Revised instruments, respectively. Scores of ≥ 25, ≥15, and ≥ 33, respectively, were considered indicative of an anxiety disorder, depressive symptoms, and a probable diagnosis of PTSD [15–20]. †Calculated using either analysis of variance or an independent sample t-test, as appropriate. ‡Using Pearson’s correlation coefficient. ^Considered statistically significant at *p* ≤ 0.05. ¶Estimated based on the participant’s age at diagnosis, disease stage, tumour histology, MYCN status (amplified versus nonamplified), and tumour cell ploidy status [21]

At T2, the univariate analysis similarly showed significant increases in anxiety (*p* = 0.001), depression (*p* < 0.001), and PTSD (*p* < 0.001) scores as age increased, with adolescents exhibiting greater (*p* = 0.006), depression (*p* = 0.001), and PTSD (*p* = 0.002) scores compared to children. Participants with a high risk of cancer had significantly higher anxiety (*p* = 0.007) and depression (*p* = 0.007) scores, while those aware of their diagnosis demonstrated significantly higher scores for anxiety (*p* = 0.007), depression (*p* = 0.003), and PTSD (*p* = 0.005). Increased anxiety scores correlated with increased depression (*p* < 0.001) and PTSD (*p* < 0.001) scores, while increased depression scores correlated with increased anxiety (*p* < 0.001) and PTSD (*p* < 0.001) scores. Finally, increased PTSD scores were associated with significant increases in both anxiety (*p* < 0.001) and depression (*p* < 0.001) scores [see Table 4].

**Table 4:** Associations between selected sociodemographic, clinical, and psychological factors and mean anxiety, depression, and post-traumatic stress disorder scores* at second investigation (T2) among Omani children and adolescents diagnosed with cancer (*N* = 92)

Characteristic	Anxiety score			Depression score			PTSD score		
	Mean ± SD	Statistics†	*p* value	Mean ± SD	Statistics†	*p* value	Mean ± SD	Statistics†	*p* value
**Gender**									
Female	22.5 ± 10.9	0.656	0.514	13.1 ± 9.6	-0.497	0.620	22.3 ± 18.0	0.481	0.631
Male	21.0 ± 11.2			14.0 ± 8.4			20.5 ± 16.0		
**Age‡**	**-**	0.424	< 0.001^	-	0.419	< 0.001^	-	0.434	< 0.001^
**Age group**									
Children	19.2 ± 8.9	-2.919	0.006^	11.3 ± 7.3	-3.529	0.001^	16.9 ± 12.8	-3.285	0.002^
Adolescents	26.9 ± 13.1			18.5 ± 10.1			30.3 ± 20.4		
**Diagnosis**									
Leukaemia	22.1 ± 10.9	0.355	0.786	15.8 ± 9.9	2.426	0.071	21.2 ± 16.1	0.872	0.459
Lymphoma	20.4 ± 9.3			9.8 ± 5.3			17.9 ± 13.4		
Solid tumour	23.1 ± 13.1			13.9 ± 9.9			25.6 ± 22.0		
Brain tumour	19.2 ± 11.4			15.2 ± 7.0			19.0 ± 10.6		
**Time since diagnosis, months**									
< 1	20.1 ± 10.2	2.501	0.088	13.0 ± 9.1	0.548	0.580	19.7 ± 17.1	2.244	0.112
1–2	26.1 ± 13.2			15.2 ± 9.6			28.4 ± 16.4		
≥ 3	24.6 ± 9.6			15.1 ± 5.1			17.3 ± 11.5		
**Type of cancer diagnosis**									
Relapse	21.8 ± 11.8	0.033	0.974	19.0 ± 10.7	1.926	0.057	22.8 ± 19.2	0.279	0.781
New	21.7 ± 11.0			13.1 ± 8.6			21.1 ± 16.7		
**Cancer risk**¶									
Not applicable	12.5 ± 4.8	4.310	0.007^	8.7 ± 4.4	4.362	0.007^	14.3 ± 8.6	2.369	0.076
Standard	19.1 ± 10.1			11.9 ± 8.7			18.2 ± 18.7		
Intermediate	19.2 ± 8.6			10.5 ± 6.2			17.1 ± 12.1		
High	25.6 ± 12.0			17.0 ± 9.8			26.2 ± 18.4		
**Cancer stage**									
Not applicable	21.7 ± 10.9	0.680	0.608	15.4 ± 10.0	1.484	0.214	21.0 ± 16.0	0.795	0.532
I	24.7 ± 11.8			10.7 ± 7.2			27.6 ± 24.2		
II	19.3 ± 9.1			10.3 ± 7.0			17.5 ± 9.4		
III	19.0 ± 11.4			10.8 ± 7.7			17.4 ± 14.9		
IV	23.9 ± 11.5			15.1 ± 8.4			24.8 ± 19.6		
**Treatment type**									
Chemotherapy alone	19.4 ± 9.3	2.090	0.089	12.2 ± 8.9	2.047	0.095	17.9 ± 13.9	2.108	0.087
Chemotherapy and radiotherapy	25.4 ± 10.3			13.7 ± 7.7			29.3 ± 21.0		
Chemotherapy and surgery	26.8 ± 13.8			14.3 ± 9.4			30.7 ± 20.5		
Chemotherapy, radiotherapy, and surgery	21.3 ± 13.3			16.5 ± 7.2			20.3 ± 15.2		
Chemotherapy and transplant	29.2 ± 16.0			22.8 ± 11.0			24.8 ± 25.8		
**Aware of diagnosis**									
Yes	27.2 ± 13.2	2.831	0.007^	18.4 ± 9.7	3.176	0.003^	30.3 ± 20.7	2.967	0.005^
No	19.4 ± 9.2			11.7 ± 7.8			17.5 ± 13.4		
**Psychological distress**									
Anxiety‡		-	-		0.550	< 0.001^		0.699	< 0.001^
Depression‡		0.550	< 0.001^		-	-		0.666	< 0.001^
PTSD‡		0.699	< 0.001^		0.666	< 0.001^		-	-

Abbreviations: PTSD: Post-Traumatic Stress Disorder, SD: Standard Deviation. *The presence of anxiety, depression, and PTSD symptoms was assessed using validated Arabic versions of the Screen for Child Anxiety Related Disorders, Center for Epidemiologic Studies Depression Scale for Children, and Impact of Event Scale-Revised instruments, respectively. Scores of ≥ 25, ≥15, and ≥ 33, respectively, were considered indicative of an anxiety disorder, depressive symptoms, and a probable diagnosis of PTSD [15–20]. †Calculated using either analysis of variance or an independent sample t-test, as appropriate. ‡Using Pearson’s correlation coefficient. ^Considered statistically significant at *p* ≤ 0.05. ¶Estimated based on the participant’s age at diagnosis, disease stage, tumour histology, MYCN status (amplified versus nonamplified), and tumour cell ploidy status [21]

A linear regression analysis was conducted to establish links between anxiety, depression, and PTSD scores and various sociodemographic, clinical, and psychological factors. At T1, significant correlations were observed between anxiety scores and age (β = 0.762; *p* < 0.001), age group (adolescents vs. children; β = -0.217; *p* = 0.001), and PTSD scores (β = 0.209; *p* = 0.025), with an adjusted R^2^ value of 0.861. Depression scores demonstrated significant correlations with age (β = 0.460; *p* = 0.001) and PTSD scores (β = 0.488; *p* < 0.001), with an adjusted R^2^ value of 0.849. Finally, PTSD scores were significantly correlated with cancer risk (β = 0.147; *p* = 0.025), anxiety scores (β = 0.287; *p* = 0.016), and depression scores (β = 0.604; *p* < 0.001), with an adjusted R^2^ value of 0.827 [see Table 5].

**Table 5:** Linear regression (enter) analysis of factors influencing anxiety, depression, and post-traumatic stress disorder scores* over time in Omani children and adolescents diagnosed with cancer (*N* = 92)

Variable	Anxiety		Depression		PTSD	
	β	*p* value	β	*p* value	β	*p* value
**First assessment (T1)**						
Age	0.762	< 0.001†	0.460	0.001†	-0.197	0.193
Age group	-0.217	0.001†	-0.113	0.091	0.119	0.107
Cancer risk‡	-	-	-0.038	0.565	0.147	0.025†
Awareness of diagnosis	0.074	0.170	-	-	0.079	0.204
Cancer stage	-	-	0.061	0.257	-	-
Anxiety score	-	-	0.098	0.391	0.287	0.016†
Depression score	0.099	0.348	-	-	0.604	< 0.001†
PTSD score	0.209	0.025†	0.488	< 0.001†	-	-
Adjusted R^2^	0.861		0.849		0.827	
**Second assessment (T2)**						
Age	0.553	< 0.001†	0.297	0.018†	-0.180	0.155
Age group	-0.134	0.014†	-0.004	0.957	0.096	0.159
Cancer risk‡	0.061	0.224	0.106	0.093	-0.039	0.541
Awareness of diagnosis	0.003	0.946	0.017	0.770	0.047	0.419
Anxiety score	-	-	0.143	0.290	0.622	< 0.001†
Depression score	0.091	0.290	-	-	0.426	< 0.001†
PTSD score	0.400	< 0.001†	0.431	< 0.001†	-	-
Adjusted R^2^	0.896		0.837		0.839	

Abbreviation: PTSD: Post-Traumatic Stress Disorder. *The presence of anxiety, depression, and PTSD symptoms was assessed using validated Arabic versions of the Screen for Child Anxiety Related Disorders, Center for Epidemiologic Studies Depression Scale for Children, and Impact of Event Scale-Revised instruments, respectively. Scores of ≥ 25, ≥15, and ≥ 33, respectively, were considered indicative of an anxiety disorder, depressive symptoms, and a probable diagnosis of PTSD [15–20]. †Considered statistically significant at *p* ≤ 0.05. ‡Estimated based on the participant’s age at diagnosis, disease stage, tumour histology, MYCN status (amplified versus nonamplified), and tumour cell ploidy status [21]

At T2, anxiety scores were found to be significantly correlated with age (β = 0.553; *p* < 0.001), age group (adolescents vs. children; β = -0.134; *p* = 0.014), and PTSD scores (β = 0.400; *p* < 0.001), with an adjusted R^2^ value of 0.896. Depression scores were significantly correlated with age (β = 0.297; *p* = 0.018) and PTSD scores (β = 0.431; *p* < 0.001), with an adjusted R^2^ value of 0.837. Finally, PTSD scores showed significant correlations with both anxiety (β = 0.622; *p* < 0.001) and depression (β = 0.426; *p* < 0.001) scores, with an adjusted R^2^ value of 0.839 [see Table 5].

## Discussion

To our knowledge, this is the first study conducted in Oman to identify the prevalence of anxiety, depression, and PTSD among Omani children and adolescents diagnosed with cancer, associated factors, and to describe changes occurring over time. Our findings revealed that a high number of children and adolescents with cancer in Oman exhibit symptoms of anxiety (43.5%), depression (56.5%), and PTSD (32.6%) within the first three months of diagnosis. Prevalence rates of these psychological disorders, especially anxiety and depression, were notably higher compared to the pooled rates reported in a recent systematic review and meta-analysis of previous literature (13.92%, 20.43%, and 20.90%, respectively) [8]. However, these differences might be attributed to variations in the measurement and screening tools used across different studies.

Alternatively, another explanation for the high prevalence rates of anxiety, depression, and PTSD symptoms observed in our study could be linked to the lack of specialized or psychosocial supportive care for cancer patients in Oman, particularly at the time of diagnosis [23]. This is likely exacerbated by the fact that, in certain Arab cultures, including in Oman, there remains considerable stigma surrounding mental health issues, posing a challenge for individuals to actively seek or obtain psychological support [24, 25]. Moreover, limitations in healthcare resources, such as a shortage of mental health professionals, may further hinder access to psychological support services for cancer patients [24]. Finally, a lack of widespread awareness regarding the significance of psychological support for cancer patients, particularly children and adolescents, could contribute to a shortage of available programs and services [26].

We also found that symptoms of anxiety, depression, and PTSD among children and adolescents diagnosed with cancer decreased over time; these findings are supported by other longitudinal studies [27–29]. Other research has shown that a healthy family environment is a strong protective factor against the development of these disorders, as well as improving the quality of life of children and adolescents diagnosed with cancer [30, 31]. In Oman, support extended by family members and friends to cancer patients has been observed to significantly reduce mental distress and alleviate the adverse side-effects associated with cancer treatment [23]. Moreover, cancer patients in Oman have been shown to develop various coping mechanisms and adaptive strategies to deal with the emotional impacts of a cancer diagnosis, including denial, optimism, withdrawal, and a strong reliance on Islamic beliefs and practices [32]. These factors likely play a role in decreasing psychological distress over time.

The results of our study indicated that the child’s age had a significant impact on their anxiety, depression, and PTSD scores, with adolescents exhibiting a higher likelihood of experiencing these conditions compared to children. Other studies have also highlighted a notable increase in major depressive episodes during the transition to adolescence [33, 34]. This finding aligns with the understanding that adolescence is marked by hormonal changes and an enhanced ability to comprehend emotions [35]. Moreover, adolescents with cancer may face substantial disruptions to their education, potentially missing school due to the demands of treatment and recovery [36]. Repercussions may extend beyond academic skills, encompassing a range of missed opportunities, such as participation in sports, group activities, excursions, and award ceremonies, as well as the absence of daily structure and routine provided in the scholastic environment [37]. Prolonged absences from school and limited peer interaction can contribute to the development of emotional, behavioural, and psychological challenges [37, 38].

We also found that children and adolescents who were informed of their diagnosis exhibited significantly higher anxiety, depression, and PTSD scores compared to those who remained unaware of their condition. The relationship between disclosure of a cancer diagnosis and mental health outcomes is complex, and individual reactions can vary widely. Some children and adolescents may benefit from being informed, as this allows them to be more actively involved in their own care and treatment decision-making, while others may find comfort in not knowing the full extent of their illness [39]. Fundamentally, awareness of a cancer diagnosis results in a deeper cognitive understanding of illness severity, the side-effects of treatment, social stigma, and health uncertainties, all of which can increase anxiety and stress [40]. However, in Omani culture, it is routine for some parents and family members to try to protect their loved ones or keep their hopes up by choosing to withhold knowledge of their diagnosis [23].

Our findings showed that high-risk patients had significantly higher PTSD scores during the first three months of diagnosis. Patients with more aggressive types of cancer often require more intensive and invasive treatment regimens, such as surgery and radiation, resulting in long periods of hospitalization, all of which may contribute to increased stress, anxiety, and trauma [41]. Furthermore, the aggressive nature of the cancer and its associated treatment can create a sense of uncertainty about the future, including treatment outcomes and the potential for relapse [42]. Indeed, the physical and emotional toll of aggressive cancer can be overwhelming as a result of the side-effects of treatment, including changes in physical appearance and disruptions to daily life, factors which can contribute to symptoms of depression [43].

The results of our study should be considered in the light of certain limitations. Firstly, the study involved a prospective, cross-sectional design in which Omani children and adolescents were screened for symptoms of anxiety, depression and PTSD at two separate time intervals following diagnosis. The length of time between these intervals might not have been adequate to track dynamic changes in anxiety and PTSD over time, thereby limiting our understanding of the long-term psychological effects of cancer diagnoses. An extended study period with more frequent assessments could have potentially enabled a more in-depth exploration of the psychological challenges faced by children and adolescents at different points in their cancer experiences.

Secondly, the information regarding anxiety, depression, and PTSD symptoms was self-assessed by the participants; such self-reporting is susceptible to various biases, including memory recall influenced by the passage of time, emotional states, and individual differences in cognitive processing. Thus, the participants may have unintentionally provided inaccurate or incomplete information regarding their psychological experiences, leading to potential discrepancies between reported and actual symptoms. Finally, we cannot rule out the effect of the confounding variables such as socioeconomic status that are associated with both the independent variable (the factor of interest) and the dependent variable (mental health outcome).

## Conclusions

To our knowledge, this is the first study conducted in Oman to identify the prevalence of anxiety, depression, and PTSD symptoms, along with their associated factors, among Omani children and adolescents diagnosed with cancer. The findings indicated that children and adolescents in Oman exhibited high levels of anxiety, depression, and PTSD within the first three months of a cancer diagnosis. Implementing routine screening protocols for psychological symptoms among children and adolescents diagnosed with cancer, particularly within the first three months of diagnosis, is imperative. The early identification of mental health challenges can facilitate timely intervention and support, particularly for adolescents, as they are more likely to suffer from psychological and emotional distress.

Furthermore, integrating mental health services into standard care protocols for paediatric and adolescent cancer patients in Oman could significantly enhance outcomes and support the delivery of holistic care. An urgent need exists for the provision of additional resources and specialised training for healthcare professionals in Oman, enabling them to recognize and address the psychological needs of children and adolescents with cancer. To advance the field, future research should consider employing longitudinal interventional designs, extending assessment durations, and incorporating a more comprehensive set of psychological variables. This approach will bolster the robustness and applicability of findings concerning mental health in the context of cancer. Additionally, longitudinal designs will enable the observation of changes in self-reported symptoms over time, offering a more nuanced understanding of the evolving psychological state of individuals navigating cancer.

## Data Availability

The datasets supporting the conclusions of this article are available from the corresponding author upon reasonable request.

## Abbreviations

CES-DC: Center for Epidemiologic Studies Depression Scale for Children
DSM-IV: Diagnostic and Statistical Manual of Mental Disorder, Fifth Edition
IES-R: Impact of Event Scale-Revised
LMICs: Low and Middle-Income Countries
MYCN: V-Myc Avian Myelocytomatosis Viral Oncogene Neuroblastoma-Derived Homolog
NOC: National Oncology Centre
PTSD: Post-Traumatic Stress Disorder
SCARED: Screen for Child Anxiety Related Disorders
SPSS: Statistical Package for the Social Sciences
SQCCCRC: Sultan Qaboos Comprehensive Cancer Care and Research Centre
SQUH: Sultan Qaboos University Hospital
